# Drug Administration Before or After Exposure to Low Temperatures—Does It Matter for the Therapeutic Effect?

**DOI:** 10.3390/ijms26083883

**Published:** 2025-04-19

**Authors:** Kadir Bezirci, Boryana Borisova, Konstantinos Papadakis, Dancho Danalev, Hristina Nocheva

**Affiliations:** 1 Department of Physiology and Pathophysiology, Medical University-Sofia, 1 Sv. Georgi Sofiyski Blvd, 1431 Sofia, Bulgaria; bezircikadir@gmail.com (K.B.); k.papadakis@medfac.mu-sofia.bg (K.P.); hndimitrova@medfac.mu-sofia.bg (H.N.); 2 Biotechnology Department, University of Chemical Technology and Metallurgy, 8 Kliment Ohridski Blvd, 1797 Sofia, Bulgaria; boriana.borisowa@gmail.com

**Keywords:** cold exposure, adaptation, pharmacokinetics, pharmacodynamics, TDIFELLK

## Abstract

The adaptation of the body when exposed to a lower-than-usual temperature is a challenge that involves neuro-endocrine–immune mechanisms and affects the pharmacokinetics and/or pharmacodynamics of drugs taken before or after cold exposure. The experiments presented in this study clearly show differences in the analgesic effect of an exogenously introduced model substance (C-terminal fragment of calcium-binding protein, spermatid-specific 1) before and after cold exposure compared to its effect at an ambient temperature. The model substance used for the experiments is an octapeptide, TDIFELLK, which was synthesized via standard solid-phase peptide synthesis. Preliminary studies proved TDIFELLK’s analgesic activity. The ANOVA analysis performed showed statistically significant differences in the pain thresholds, measured by a paw pressure test, in 109 rats distributed among 14 groups and subjected to cold exposure according to different set-ups. Cold exposure immediately after TDIFELLK administration appears to enhance its analgesic effect, while cold exposure before administration reduces the effect. In some of the set-ups, antagonists of the most significant for analgesia receptors, i.e., opioid, cannabinoid, and serotonergic, were also introduced. The results showed that cold exposure had a modulating influence on the effect of the exogenously administered substances. The modulating effect was manifested differently depending on whether the intake occurred before or after cold exposure. The results also showed that the interaction with individual mediator systems was also subjected to differences depending on intake occurring before and after cold exposure.

## 1. Introduction

Ambient temperature is among the most significant factors that directly and imperatively affect the internal continuum, and hence the vital functions via continuous environment–organism interaction. Environmental temperature and its influence on organisms were initially given attention due to the potential effects of sudden temperature changes on agricultural crops, yield, and livestock [1]. The impact of significantly high or low temperatures on the human body and the possible pathological effects of such exposure have become the subject of attention mainly in relation to observed occupational diseases and sports [2,3]. However, a relatively limited number of studies show that low temperatures have serious consequences for the human body’s motor, respiratory, urinary, nervous, and other systems, as well as homeostasis in general. Among the most eloquent examples is the increased risk of a coronary artery spasm at low ambient temperatures. This event is immediately triggered by low temperatures due to short-term regulatory mechanisms affecting cardiovascular and cardiac autonomic control, accompanied by changes in heart rate and blood pressure [4,5]. Some other examples are the increased incidence of respiratory diseases during cold months, joint pathologies, etc. [6,7]. A lack of timely and adequate treatment for acute cold exposure could aggravate the original trauma, with an extremely high mortality rate [8].

The body could randomly be exposed to low temperatures as the result of an accident or consciously. Thus, the need for more in-depth research on the mechanisms of impact of low temperatures on the body arises. The latest opportunities to peer at molecular-level cellular functions have made it possible to establish the cold-inducible RNA-binding protein (CIRP). Initially discovered in 1997 in mouse germ cells [9], in humans, the CIRP modulates the translational aspects of gene expression, including mRNA splicing, stability, and transport, in an attempt to shield cells from the damaging effects of low temperatures. The discovery that the CIRP can affect gene expression shows that body exposure to low temperatures can have pathological consequences not only for individual systems, but for the entire organism [10,11].

Our previous studies, as well as the literature data, show that various stressogenic factors, including low temperatures, trigger neuro-endocrine–immune mechanisms for the purpose of organism adaptation to changes in the environment [12]. People often consume different substances, such as drugs and supplements, for prevention. The process of substance intake could be realized in conditions of cold exposure (CE). Thus, it seems logical to ask whether the processes aimed at restoring the body’s homeostasis will affect such exogenously administered substances. In order to prove or disprove such changes in the effect, a series of studies with the exposure of rats to low temperature was conducted. The introduction of the exogenous substance was conducted immediately before or after CE. To prove possible mechanisms of the monitored effects, antagonists of the most important for analgesia receptors (opioid, cannabinoid, and serotonergic) were used.

The choice of a suitable substance for this research was made based on the following two criteria: (1) to possess an effect that can be relatively easily quantified (in our study, the chosen effect is analgesia, which is relatively easily monitored via the paw pressure test) and (2) to resemble naturally occurring molecular sequences in the body to ensure that the body has well-conditioned pathways for its intermediate exchange. Herein, this substance with a proven analgesic effect was used in order to avoid any conflicts of interest.

The chosen substance was described by Laurent et al. Many years ago, they studied a highly expressed protein in the salivary glands of rats named protein submandibular rat 1 (SMR1) [13]. This acts as a prohormone containing the amino acid sequence TDIFEGG near the C-terminus. It was found that this heptapeptide had many positive effects on the organism, such as inflammation reduction and the improvement of outcomes in preclinical models of allergic asthma, lung injury, etc. [14,15,16,17,18,19]. Additionally, Ritz et al. found that in humans, there is a calcium-binding protein, spermatid-specific 1, which has a C-terminal heptapeptide TDIFELL analogous to the TDIFEGG sequence of SMR1 [20]. The in vivo tests performed by Laurent et al. showed the enlargement of the C-terminus in TDIFELL and TDIFELLK was more promising than that of the parent molecule since it expressed better physiological properties [13]. Encouraged by the positive results revealed by Laurent et al., herein, TDIFELLK was used in order to study the analgesic effect when it is introduced to the body at a normal environmental temperature and in conditions of CE.

The goals set are as follows:

(1) To compare the analgesic effects of TDIFELLK after administration at a normal temperature and in the settings of CE using two administration options—(a) before and (b) after CE;
(2) To compare the effects of TDIFELLK after antagonizing the following:
  - Opioid receptors with naloxone;
  - Cannabinoid receptor type 1 (CB1) with N-(Piperidin-1-yl)-5-(4-iodophenyl)-1-(2,4-dichlorophenyl)-4-methyl-1H-pyrazole-3-carboxamide AM251;
  - Serotoninergic receptor 1A (5HT1A) with NAN-190 hydrobromide (NAN).

Similarly, the effect was determined after administration at a normal temperature and in the settings of CE again according to the above two administration options: before and after CE.

## 2. Results

**Figure 1:**
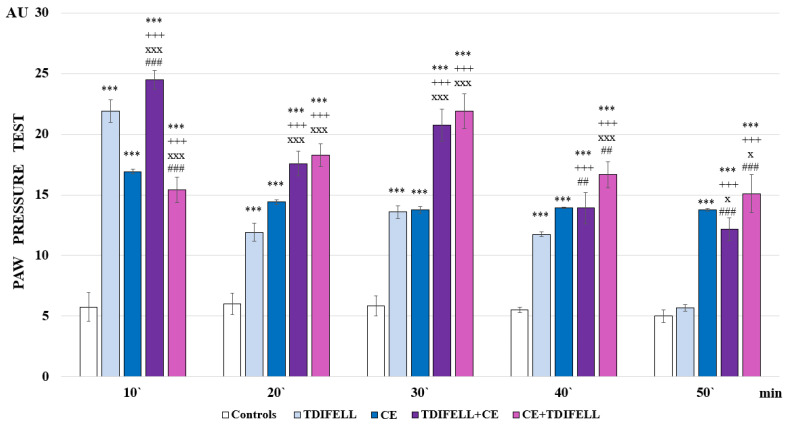
Effects of TDIFELLK administration without and with (before/after) 1 h of cold exposure (CE) estimated by PP test in rats. Mean values ± S.E.M. are presented in arbitrary units (AU) at the 10th, 20th, 30th, 40th, and 50th min of the experiment. *** *p* < 0.001 vs. controls; TDIFELLK + CE and CE + TDIFELLK have been compared vs. TDIFELLK (^+++^ *p* < 0.001) and vs. CE (^xxx^ *p* < 0.001; ^x^ *p* < 0.05); TDIFELLK + CE vs. CE + TDIFELLK (^###^ *p* < 0.001; ^##^ *p* < 0.01).

An hour of CE statistically significant increases the PP thresholds of the animals compared to the control group during the whole experiment (F_1,12_ = 626.42453; *p* < 0.00001 at the 10th min; F_1,12_ = 296.69552; *p* < 0.00001 at the 20th min; F_1,12_ = 517.23077; *p* < 0.00001 at the 30th min; F_1,12_ = 1336.5; *p* < 0.00001 at the 40th min; F_1,12_ = 1126.23077; *p* < 0.00001 at the 50th min) (Figure 1).

Introduced alone and without CE, TDIFELLK shows a statistically relevant analgesic effect compared to the control group for the first 40 min of the experiment (F_1,13_ = 971.79718; *p* < 0.00001 at the 10th min; F_1,13_ = 105.7273; *p*< 0.00001 at the 20th min; F_1,13_ = 402.7268; *p* < 0.00001 at the 30th min; F_1,13_ = 642.85714; *p*< 0.00001 at the 40th min). The graphic of PP thresholds formed a U-shaped curve with peaks at the 10th and 30th min. After the 40th min, the PP thresholds abruptly decreased to those of the control group (Figure 1).

The administration of TDIFELLK in terms of CE leads to some statistically relevant differences in the described effects. TDIFELLK administered prior to CE (TDIFELLK + CE group) expresses at the 10th min a statistically relevantly higher analgesic effect compared to TDIFELLK alone (F_1,15_ = 38.58015; *p* = 0.000023), group one after 1 h in CE (F_1,14_ = 632.59175; *p* < 0.00001), and TDIFELLK administered after CE (CE + TDIFELLK group) (F_1,15_ = 454.42222; *p* < 0.00001). This initial increase in analgesia was brief. For the rest of the time, the effect shown by the TDIFELLK + CE group was comparable (at the 20th and 30th min) and statistically relevantly lower than the CE + TDIFELLK group at the 40th (F_1,15_ = 16.1563; *p* = 0.001267) and 50th min (F_1,15_ = 22.76984, *p* < 0.000298). The graphic of PP thresholds formed a U-shaped curve with peaks at the 10th and 30th min, resembling that of the TDIFELLK group described in Figure 1. However, if TDIFELLK was introduced after the animals were removed from the cold chamber, initially (at the 10th min) the substance presented with lower PP thresholds compared to administration alone or before CE, and comparable to CE alone. An increase in the analgesic effect was observed until the 30th min, followed by a gradual decrease (Figure 1).

**Figure 2:**
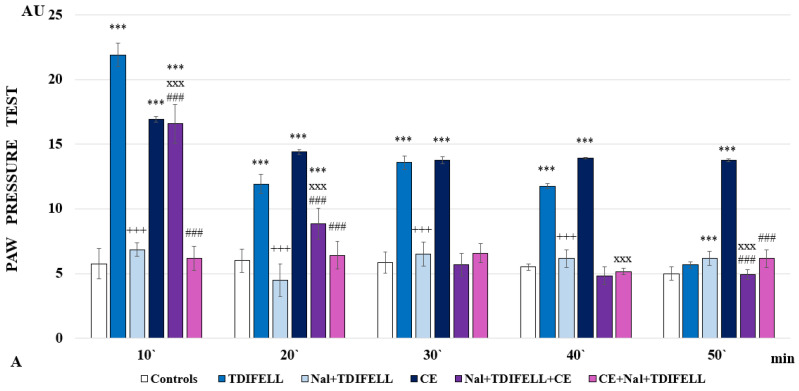

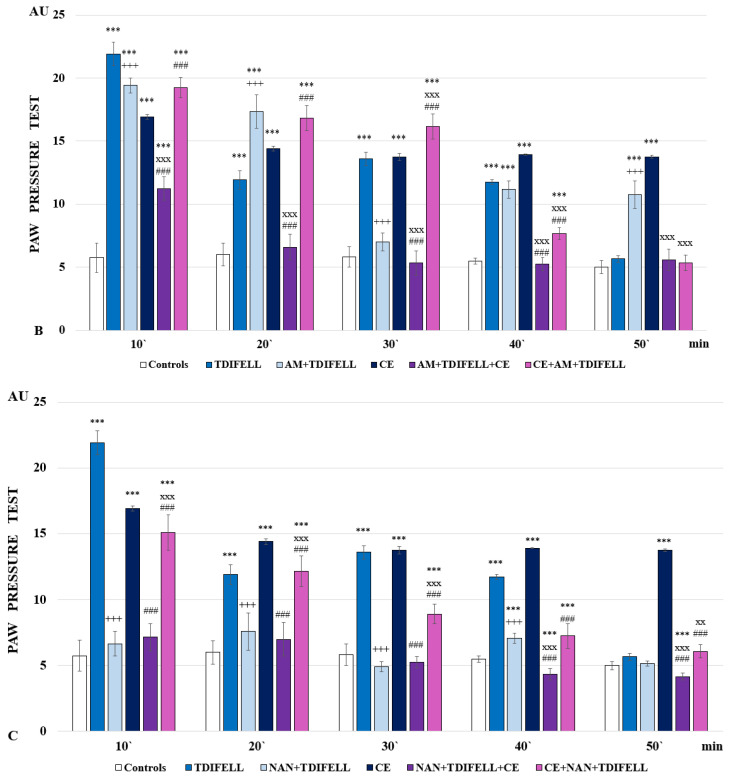
Effects of TDIFELLK administration without and with (before/after) 1 h of cold exposure (CE) estimated by PP test in rats after antagonization of (**A**) opioid receptors with naloxone: *** *p* < 0.001 vs. controls; Nal + TDIFELLK vs. TDIFELLK ^+++^ *p*< 0.001 vs.; Nal + TDIFELLK + CE and CE + Nal + TDIFELLK vs. Nal + TDIFELLK ^xxx^ *p* < 0.001; Nal + TDIFELLK + CE vs. CE + Nal + TDIFELLK ^###^ *p* < 0.001; (**B**) cannabinoid receptor type 1 with AM251: *** *p* < 0.001 vs. controls; AM + TDIFELLK vs. TDIFELLK ^+++^ *p* <0.001 vs.; AM + TDIFELLK + CE and CE + AM + TDIFELLK vs. AM + TDIFELLK ^xxx^ *p* < 0.001; and AM + TDIFELLK + CE vs. CE + AM + TDIFELLK ^###^ *p* < 0.001; (**C**) 5HT1A receptor with NAN-190: *** *p* < 0.001 vs. controls; NAN + TDIFELLK vs. TDIFELLK ^+++^ *p* < 0.001; NAN + TDIFELLK + CE and CE + NAN + TDIFELLK vs. NAN + TDIFELLK ^xxx^ *p* < 0.001; ^xx^ *p* < 0.01; NAN + TDIFELLK + CE vs. CE + NAN + TDIFELLK ^###^ *p* < 0.001. Mean values ± S.E.M. are presented in arbitrary units (AU) at the 10th, 20th, 30th, 40th, and 50th min of the experiment.

The administration of TDIFELLK without CE along with the opioid receptor antagonist naloxone completely prevents the development of analgesia, with even a trend toward hyperalgesia monitored at the 20th min. A similar effect is observed when the combination Nal + TDIFELLK is introduced after CE (CE + Nal + TDIFELLK group), but the PP values are comparable to controls throughout the follow-up period, without registering a trend towards hyperalgesia. The administration of Nal + TDIFELLK prior to CE (Nal + TDIFELLK + CE-group) shows at the 10th min PP values comparable to those of the CE group. A lowering of PP thresholds is registered at the 20th min, and from the 30th min they remain comparable to the control ones. The important finding is that during the first 20 min and at the 50th min, the analgesic effect of the Nal + TDIFELLK + CE group and the CE + Nal + TDIFELLK group are statistically relevantly different from each other (F_1,15_ = 384.24701; *p* < 0.00001 at the 10th min; F_1,15_ = 23.95261; *p* = 0.000237 at 20th min; F_1,15_ = 25.52525; *p* = 0.000177 at the 50th min) (Figure 2А).

The reported analgesia of TDIFELLK without CE appears to be less dependent on cannabinoid receptors. Antagonizing CB1 with AM251 lowers PP-thresholds to control values only at 30th min; during the remaining time, the analgesia reported is comparable (at the 10th and 40th min) or even exceeds (at the 20th and 50th min) that of the TDIFELLK group. Administration of the combination AM251 + TDIFELLK after 1 h CE (CE + AM + TDIFELLK group) increases the PP thresholds compared to those of the CE group during the first 30 min. Further, the thresholds drop sharply and at the 50th min their values are comparable to those of controls. On the contrary, the introduction of the combination AM251 + TDIFELLK before CE (AM + TDIFELLK + CE group) reduces the PP values as early as the 10th min, and from the 20th min the PP thresholds are comparable to the control ones. The important finding is that during the first 40 min, the analgesic effects of the AM + TDIFELLK + CE group and CE + AM + TDIFELLK group are statistically relevantly different from each other (F_1,15_ = 448.85129; *p* < 0.00001 at the 10th min; F_1,15_ = 352.56929; *p* < 0.00001 at the 20th min; F_1,15_ = 332.18182; *p* < 0.00001 at the 30th min; F_1,15_ = 74.45455; *p* < 0.00001 at the 40th min) (Figure 2В).

Antagonizing the 5-HT1_A_ receptor with NAN-190 without CE prevents the development of analgesia from the beginning of the experiment and throughout the follow-up period. The NAN-190 + TDIFELLK combination introduced before CE (NAN + TDIFELLK + CE group) also prevents the development of analgesia as early as the 10th min, with a trend towards hyperalgesia being noted from the 30th min onwards. Conversely, administration of the same combination after CE (CE + NAN + TDIFELLK group) induces analgesia throughout the follow-up period. Thus, the important finding is that during the whole time the analgesic effect of NAN + TDIFELLK + CE group and CE + NAN + TDIFELLK group are statistically relevantly different from each other (F_1,15_ = 236.45106; *p* < 0.00001 at the 10th min; F_1,15_ = 98.88235; *p* < 0.00001 at the 20th min; F_1,15_ = 204.76471; *p* < 0.00001 at the 30th min; F_1,15_ = 97.25157; *p* < 0.00001 at the 40th min; F_1,15_ = 61.76471; *p* < 0.00001 at the 50th min) (Figure 2С).

## 3. Discussion

The initial hypothesis that CE would alter the effect of an exogenously administered model substance was confirmed by the obtained results. The realized experiments indeed showed that the effect of the TDIFELLK alone and without CE differs in intensity and duration from that after its introduction before/after CE. The explanation for the monitored effects could be sought in the changes in the pharmacokinetics (PhК) and pharmacodynamics (PhD) of the substance under conditions of CE. Cold challenge, like any stressor, involves adaptation consisting in a new equilibrium in the level of stress hormones and other physiological responses [21]. It could be assumed that different types of receptors, or mediators, are involved in the effects, and their “activation” occurs at a different stage, which is why the effect fluctuates over time and an interesting U-shaped effect is monitored. However, this hypothesis will be a subject for further clarification.

**Figure 3:**
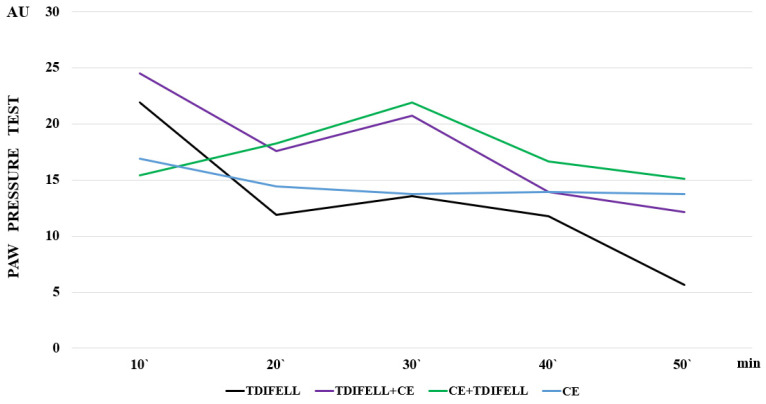
The effect curves are presented, illustrating the differences in the effect of cold exposure (СЕ) without the substance (blue line), and TDIFELLK administered at normal temperature (black line), before (purple line) and after (green line) СЕ.

One of the first and obligatory changes when the body is exposed to cold is an increase in resting metabolic rate, in order to maintain the body’s temperature homeostasis [22,23,24,25]. Partially, this readjustment of metabolic processes is related to the redistribution of blood flow under conditions of various types of stress, in order to ensure the most adequate blood supply to vital organs [26]. The redistribution of blood in the body would also affect the distribution of an exogenously introduced substance. In our experiments, for example, the increased effect of TDIFELLK, introduced immediately before CE, could be due to a delay in the metabolism in the body because of the impaired distribution. The introduction of the substance immediately before CE suggests that its metabolism will coincide with the adaptation processes that the low temperature will trigger. Conversely, the introduction of TDIFELLK after the end of CE suggests that its metabolism will coincide with the processes of restoring normal homeostasis. This is a possible explanation for the differences observed in our results in the effect curves of TDIFELLK at normal temperature, before and after CE (Figure 3).

At the same time, Figure 3 shows that for a short time between the 20th and 30th min, the slopes of the TDIFELLK curves before and after CE are similar. Further, they become statistically relevantly different again. Such effects resemble a reflection of the effect of cold on the PhK of the substance due to its distribution, but also absorption, metabolism, or excretion.

Initially, in order to preserve heat in the body, vasoconstriction is observed, best manifested in the skin. But it can also affect the gastrointestinal tract, thus affecting the absorption of drugs administered orally, transdermally, or subcutaneously. Vasoconstriction could also affect the distribution of the drug in different parts of the body, as well as binding to plasma proteins (determining the free, active fraction, and the bound, inactive one) [27,28].

Enhanced energy metabolism for the needs of thermogenesis demands nearly 40% of cardiac oxygen to be delivered to skeletal muscles and liver [29]. The facultative thermogenesis in humans primarily occurs in the brown adipose tissue (BAT) and skeletal muscle, which should be considered (for drugs with intramuscular administration) [30]. The increased basic metabolism in the liver, considering its central role in the so-called “first pass exchange” of drugs and in their subsequent processing in the body, changes liver enzymes’ activity (e.g., cytochrome P450 system) and affects the PhK of drugs—delaying their inactivation [31,32]. In our experiments, such changes in metabolic rate could underline the delay in the effect at the 10th min of TDIFELLK administered after CE, followed by an increase compared to the effect of CE itself and the effect of the substance without thermal stress (Figure 1).

Cold-induced diuresis is also known, which is due to the neuroendocrine effects of cold on kidney epithelial tissue [33,34]. If it is assumed that the increased diuresis, induced by CE, leads to relative hypovolemia, then the concentration of TDIFELLK administered immediately before CE has to be increased. Do not forget that the substance has just been introduced; therefore, it has not yet been metabolized, and thus its excretion will be lower. This may explain the increased effect of the substance at the 10th min. The normalization of metabolism after the end of CE also normalizes TDIFELLK’s metabolism, which leads to a faster decrease in its effect compared to that of TDIFELLK taken after the end of CE. Interestingly, the same U-shape pattern of the analgesic effect is noted as that described for TDIFELLK administration alone, probably due to the time-dependent participation of different receptors.

Currently, the full effects of mild CE on the vital organs are not completely characterized. However, cold adaptation is the body’s protective response, and the molecular mechanisms underlying this response in mammalian cells are just beginning to be understood [35,36].

**Figure 4:**
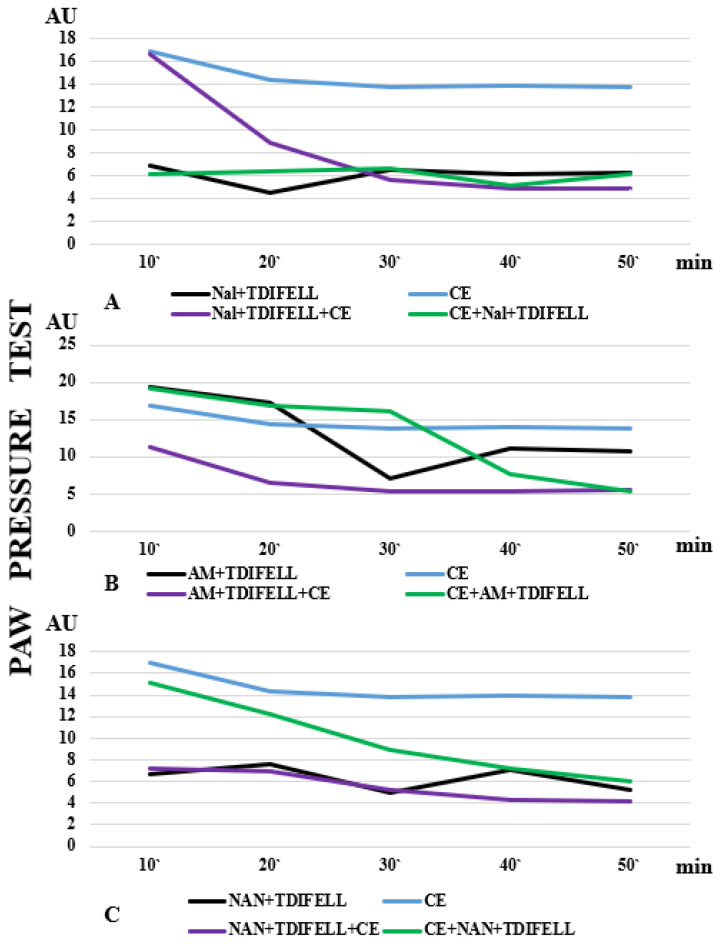
The effect curves after antagonizing (**A**) opioid, (**B**) cannabinoid CB1, and (**C**) 5HT1A receptors are presented. The differences in analgesia induced by cold exposure (CE) alone (blue line), the administration of TDIFELLK with the corresponding antagonist at normal temperature (black line), and the administration of TDIFELLK with the corresponding antagonist before (purple line) and after (green line) CE are clearly visible.

Thus far, potential changes in PhK that could explain the results have been discussed. We believe that the results related to receptor antagonism’s study are likely to be due to the influence of PhD to a greater extent. The binding of a substance to a receptor depends on the expression of the receptor and its affinity as well as the concentration of the substance and the affinity to the receptor. A greater dependence of an effect on a particular receptor suggests that antagonizing the receptor would significantly reduce the effect of the substance. The performed experiments showed а distinct dependence (manifested as prevention) of TDIFELLK’s analgesic effect on the opioid and 5-HT1A receptors, whose antagonism completely prevented the development of analgesia. The introduction of the combination Nal + TDIFELLK immediately before CE, when their metabolism would coincide with the adaptation processes, changed the dependence of the effect on the opioid receptors. The analgesia reported at the 10th and 20th min could be due to the delayed binding of naloxone to the receptors. Stress is known to increase the release of endogenous opioid receptor ligands, whose binding could delay the binding of naloxone [37]. Another explanation could be that cold affects membrane structures (mainly lipids). In addition, the cold changes their fluidity, and hence binding, as well as the activity of drugs that interact with membrane-bound receptors or channels [38]. Cold adaptation can also modify intracellular signaling pathways, potentially affecting the response to drugs that act on these pathways (e.g., cannabinoid receptor mediators are synthesized on demand, which means that their synthesis, and hence the effect, can be influenced by the general change in metabolism during CE). There is no delay in antagonizing analgesia with naloxone and TDIFELLK after the end of CE. It is likely that in this case the receptor is already “free” to bind naloxone. The described differences in the effect of naloxone when administered before/after CE are extremely well illustrated by the presentation of the effect curves (Figure 4). The blue line in Figure 4A shows the analgesia induced by CE. The black line shows the complete lack of analgesia after opioid receptor antagonism by the combination Nal + TDIFELLK at room temperature. The purple line shows a similar lack of analgesia with receptor antagonism immediately after the end of CE. The green line shows the initial analgesia due to the delayed effect of naloxone (Figure 4А).

The graphs shown in Figure 4 clearly illustrate the differences in the effect curves of TDIFELLK after antagonizing the other two types of receptors—CB1 (Figure 4B) and 5HT1A (Figure 4C). The differences in the effects without and with CE indicate that low temperature alters the effect of the substance to some extent. It has to be clearly emphasized that this is not about obtaining the opposite effect (TDIFELLK induced analgesia in all experimental settings), but the degree of this effect differs. The effect has been modulated. Similarly, the effect of antagonizing different types of receptors was also modulated.

The relationship of the specific mediator system to the PhK and PhD of the drug is probably also important. For example, regarding the combinations Nal + TDIFELLK and AM251 + TDIFELLK, it turns out that the effect of their application after CE is similar to that without CE. It could be speculated that when the combinations were injected immediately before CE, the interaction of TDIFELLK with the opioid, respectively, CB1 receptors, coincides with cold adaptation, which significantly changes the PhK and PhD of TDIFELLK. The same “logic” is not valid for the NAN-190 + TDIFELLK combination. This could be due to the different (more secondary) involvement of the serotonergic system in the mechanisms of body adaptation to stress and analgesia (while opioid and cannabinoid receptors have a major role) [39,40].

Cellular and molecular mechanisms of adaptation are also important. Cold induces the expression of stress proteins, e.g., heat shock proteins [35], which may interact with drug targets or influence cellular responses to drugs. It is known that moderate CE increases CIRP in rat brains. At the same time, data exist that CIRP induces TRX in the brain, liver, heart, muscles, and BAT of rats [41,42,43]. Thus, additional positive relationships exist that may deepen/prolong the effect on total metabolism, and hence on the PhK and PhD of drugs taken in relation with a CE.

At the same time, “local” manifestations of the drug/cold/organism interaction are possible. For example, increased heart rate and arterial pressure (resulting from the general effect of cold on the body through the autonomic nervous system involved in adaptation to low temperatures) can change the effect of cardiovascular drugs (e.g., antihypertensives, beta-blockers). Since the role of low environmental temperatures for cardiovascular pathology is well known, there are pharmacotherapeutic agents for the treatment and prevention of specific cold-induced conditions [44,45]. But the impact of CE on the drugs themselves has not been studied. The effect of agents affecting the immune system may also be specifically altered due to the suppressive/activating effect of cold on the immune system. Cold stress has been reported to sensitize the neuro-immune reactivity in the rat brain [46]. Any such exposure can have an adverse effect on humans.

The current study also has some logical limitations. For example, experiments were conducted on animals, not on humans. This also determines some of the possible further directions, e.g., conducting similar studies on workers at low temperatures. In these cases, it would be appropriate to combine the study of the in vivo effect with the monitoring of differences in some indicators which would suggest potential changes in the PhK of the substance, for example, the plasma concentration of the substance, urinary excretion, and body temperature. The possibility of monitoring PhD’s parameters such as receptor expression, affinity, and reuptake, as well as plasma levels of specific mediators, would also be of interest.

To summarize the results of the experiments, it can be concluded that (1) cold exposure changed the effect of the introduced TDIFELLK; (2) the change in the effect differed depending on whether the substance was introduced before or after CE; (3) antagonizing different types of receptors had different effects on analgesia without and with CE; (4) the change in effect again differed depending on whether the introduction was before or after CE. The study represents an obvious visual demonstration that “physiological” changes in the organism’s metabolism (e.g., thermogenesis and energy metabolism) modify the effect of exogeneously administered substances compared to their administration at normal temperature. It could be assumed that these changes are due to the effects on a drug’s PhK and PhD, with potential modification of the therapeutic effect and most likely also side effects. The dependence of the drug effect on the ambient temperature should be taken into account in cases where a pharmacotherapeutic approach is required in individuals abruptly exposed to low temperatures. The study of the specific organism/drug interaction under conditions of CE is necessary in view of the optimization of the molecular design of newly synthesized substances with potential therapeutic applications. An example could be predicting the need for the dose adjustment of certain medications in patients who have suffered frostbite or are working in low temperatures, for example, hypertensives, diabetics, hemophiliacs, etc., who are not disabled but need to take certain medications regularly. It is also necessary to study the changes in individual mediator systems during CE, as they determine to a significant extent the final effect of drugs’ administration. This is particularly important for medicinal products whose targets are systems with a more intense participation in stress adaptation: the cardiovascular, respiratory, nervous, and immune systems.

## 4. Materials and Methods

### 4.1. Animals

The experiments were carried out on male Wistar rats (180–200 g), obtained from the vivarium of the Medical University of Sofia, Bulgaria. The animals were group-housed in polypropylene cages (40 × 60 × 20 cm) in a temperature-controlled colony room maintained at 21 ± 3 °C under a 12 h light/12 h dark cycle with lights on at 8:00 a.m. [47,48]. The reference used is “Normal” temperature 21 ± 3 °C.

Free access to water and standard rat chow was allowed. The experiments were carried out between 9.00 and 12.00 a.m.

After a week of acclimatization, rats were randomly assigned to different groups: a control group (*n* = 6) and 13 experimental groups (*n* = 7–8), detailed in Table 1 [49,50].

All procedures were approved by the Animal Care and Use Committee of the Medical University of Sofia, and permission from BAFS has also been issued (No. 353/14.06.23).

### 4.2. Acute Cold Exposure (CE) Protocol

The rats in the experimental groups were transported in their home cages, with food, water, and bedding, into a temperature-controlled chamber and exposed to 4 °C for 1 h [47,48].

The rats in the control group were housed at normal temperature during this period.

### 4.3. Drugs and Treatment

The CB1-agonist N-arachidonoyl-ethanolamine (AEA, 1 mg/kg BW); the CB1-antagonist N-(Piperidin-1-yl)-5-(4-iodophenyl)-1-(2,4-dichlorophenyl)-4-methyl-1H-pyrazole-3-carboxamide (AM251, 1.25 mg/kg BW); naloxone (Nal, 1 mg/kg BW); and the 5HT1_A_-antagonist NAN-190 hydrobromide (NAN, 1 mg/kg BW), were dissolved in the vehicle and intraperitoneally administered in different combinations before or after cold exposure, according to the descriptions in Table 1 [51,52].

### 4.4. Peptide Synthesis

The CABS1 C-terminal moiety TDIFELLK was synthesized using a standard manual solid-phase peptide synthesis, Fmoc/Ot-Bu strategy. Briefly, the peptide was synthesized on 2-chlorotrityl chloride resin using diisopropylcarbodiimide as a coupling reagent (Iris Biotech, Wunsiedel, Germany) by sequentially linking the successively protected amino acids of the primary structure of the peptide, growing the peptide chain from the C- to the N-terminus. The final targeted peptide was deprotected and released from the resin by treatment with a mixture of 50% TFA/50% dH_2_O:TIS (97.5:2.5 eq.) for 4 h. The raw TFA extract of peptide was crystalized by adding of a cold diethyl ether to the obtained oil. TDIFELLK was synthesized with 63% of yield, M.p. 213–215 °C, [α]_D_^20^ = −15 (c = 1, DMSO). The chromatographic purity of the targeted peptide was 96%.

### 4.5. Paw-Pressure Test (Randall–Selitto Test)

The changes in the mechanical nociceptive threshold of the rats were measured by an analgesimeter (Ugo Basile, Gemoni, Italy). The pressure was applied to the hind-paw and the weight (in arbitrary units, AU) required to elicit a nociceptive response (squeak or struggle) was taken as the mechanical nociceptive threshold (paw pressure threshold, PPT). A cut-off value of 500 g (25 AU) was used to prevent damage to the paw [53].

### 4.6. Data Analysis

The results were statistically assessed by a one-way analysis of variance (ANOVA) followed by a Tukey HSD comparison test [47]. Values were presented as the mean ± S.E.M; *p* < 0.05 was considered to indicate statistical significance.

## 5. Conclusions

The environmental temperature impacts animals’ physiology and behavior; thus, exposure of the body to mild acute cold stress may act as a metabolic stressor, and by changing the PhK and PhD of exogenous (and possibly endogenous) substances, it can modulate their potential beneficial (and/or side) effects. In our studies, the introduction of the exogenous substance TDIFEELK before cold exposure showed a stronger analgesic effect compared to that after the end of cold exposure. We believe that the possible underlying mechanisms for these changes are differences in the intermediate metabolism of the body due to the cold-induced adaptation mechanisms. Also, our studies on the effect of antagonizing opioid, CB1, and 5HT1A receptors showed that cold exposure likely alters their affinity and binding to antagonists. Studying specific interactions between drugs and mediator systems involved in their effect would allow for the creation of individualized regimens and dosages for patients whose occupational activity includes exposure to low temperatures.

## Figures and Tables

**Table 1:** Treatments of animals from the different experimental groups.

No	Group	Treatment
1	Control	This group was not exposed to cold and was treated with saline
2	TDIFELLK	Animals in the group were injected intraperitoneally (i.p.) with TDIFELL (2 mg/kg)
3	CE	Animals in the group were exposed to cold for 1 h (1 h) in a cold chamber (4 °C)
4	TDIFELLK +CE	Animals in the group were injected i.p. with TDIFELL (2 mg/kg), after which they were exposed to 1 h CE
5	CE + TDIFELLK	Animals in the group were exposed to 1h CE, after which they were injected i.p. with TDIFELL (2 mg/kg)
6	Nal + TDIFELLK	Animals in the group were injected i.p. with the combination naloxone (Nal, opioid receptors antagonist; 1 mg/kg) and TDIFELL (2 mg/kg)
7	Nal + TDIFELLK + CE	Animals in the group were injected i.p. with the combination Nal (1 mg/kg) + TDIFELL (2 mg/kg), after which they were exposed to 1 h CE
8	CE + Nal + TDIFELLK	Animals in the group were exposed to 1 h CE, after which they were injected i.p. with the combination Nal (1 mg/kg) + TDIFELL (2 mg/kg)
9	AM + TDIFELLK	Animals in the group were injected i.p. with the combination АМ251 (AM, cannabinoid receptor type 1 antagonist; 1.25 mg/kg) + TDIFELL (2 mg/kg)
10	AM + TDIFELLK + CE	Animals in the group were injected i.p. with the combination AM (1.25 mg/kg) + TDIFELL (2 mg/kg), after which they were exposed to 1 h CE
11	CE + AM + TDIFELLK	Animals in the group were exposed to 1 h CE, after which they were injected i.p. with the combination АМ (1.25 mg/kg) + TDIFELL (2 mg/kg)
12	NAN + TDIFELLK	Animals in the group were injected i.p. with the combination NAN-190 (NAN, 5HT1A receptor antagonist; 1 mg/kg) + TDIFELL (2 mg/kg)
13	NAN + TDIFELLK + CE	Animals in the group were injected i.p. with the combination NAN (1 mg/kg) + TDIFELL (2 mg/kg), after which they were exposed to 1 h CE
14	CE + NAN + TDIFELLK	Animals in the group were exposed to 1 h CE, after which they were injected i.p. with the combination NAN (1 mg/kg) + TDIFELL (2 mg/kg)

## Data Availability

Тhe results published in this article are part of the currently developing Ph.D. theses of Boryana Borisova and Kadir Bezirci. Thus, they are available from the heads of both Ph.D. students, D.D. and H.N.

## Abbreviations

AU	arbitrary units
BAT	brown adipose tissue
CE	cold exposure
CABS1	calcium-binding protein, spermatid-specific 1
CIRP	cold-inducible RNA-binding protein
PD	pharmacodynamics
PK	pharmacokinetics
ROS	reactive oxygen species
SMR1	protein submandibular rat 1
TRX	thioredoxin
TIS	triisopropylsilane
TFA	trifluoroacetic acid
